# Deciphering the interaction of bacteria inoculants with the recipient endophytic community in grapevine micropropagated plants

**DOI:** 10.1128/aem.02078-23

**Published:** 2024-01-30

**Authors:** Lorenzo Vergani, Joa Patania, Valentina Riva, Luca Nerva, Floriana Nuzzo, Giorgio Gambino, Sara Borin, Francesca Mapelli

**Affiliations:** 1 Department of Food, Environmental and Nutritional Science (DeFENS), University of Milan, Milan, Italy; 2 Institute for Sustainable Plant Protection, National Research Council of Italy (IPSP-CNR), Turin, Italy; 3 Italy Research Centre for Viticulture and Enology, Council for Agricultural Research and Economics, Conegliano, Italy; Michigan State University, East Lansing, Michigan, USA

**Keywords:** plant microbiome, plant growth-promoting bacteria, bacterial invasion, plant micropropagation, endosphere, *Rhizobium*

## Abstract

Engineering the plant microbiome with beneficial endophytic bacteria can improve the growth, health, and productivity of the holobiont. Here, we administered two beneficial bacterial strains, *Kosakonia* VR04 sp. and *Rhizobium* GR12 sp., to micropropagated grapevine cuttings obtained via somatic embryogenesis. While both strains colonized the plant endosphere, only *Rhizobium* GR12 sp. increased root biomass under nutritional-deficit conditions, as supported by the plant growth promotion traits detected in its genome. Phylogenetic and co-occurrence analyses revealed that the plant native bacterial community, originally dominated by Streptococcaceae and Micrococcaceae, dramatically changed depending on the inoculation treatments, as invading strains differently affected the relative abundance and the interactions of pre-existing taxa. After 30 days of plantlets’ growth, *Pantoea* became a predominant taxon, and considering untreated plantlets as references, *Rhizobium* sp. GR12 showed a minor impact on the endophytic bacterial community. On the other hand, *Kosakonia* sp. VR04 caused a major change in community composition, suggesting an opportunistic colonization pattern. Overall, the results corroborate the importance of preserving the native endophytic community structure and functions during plant microbiome engineering.

**IMPORTANCE:**

A better comprehension of bacterial colonization processes and outcomes could benefit the use of plant probiotics in the field. In this study, we applied two different beneficial bacteria to grapevine micropropagated plantlets and described how the inoculation of these strains impacts endophytic microbiota assembly. We showed that under nutritional deficit conditions, the response of the receiving endophytic bacterial communities to the invasion of the beneficial strains related to the manifestation of plant growth promotion effects by the inoculated invading strains. *Rhizobium* sp. GR12 was able to preserve the native microbiome structure despite its effective colonization, highlighting the importance of the plant-endophyte associations for the holobiont performance. Moreover, our approach showed that the use of micropropagated plantlets could be a valuable strategy to study the interplay among the plant, its native microbiota, and the invader on a wider portfolio of species besides model plants, facilitating the application of new knowledge in agriculture.

## INTRODUCTION

Endophytes are considered the most interesting among beneficial microbiome members, as they can interact intimately with plants, penetrating their internal tissues and moving in different compartments (1). These capabilities imply that endophytic strains can be highly efficient as plant probiotics since (i) they are less exposed to the variation of physicochemical parameters occurring in the outer plant environments (i.e., leaf surface, rhizoplane, and rhizosphere) and (ii) they suffer a minor competition in the endosphere compared to other microhabitats (2, 3).

The occurrence of a plant growth promotion (PGP) effect after the application of a beneficial bacterium, generally defined by measuring phenotypic traits, may depend upon several factors, including the bacteria’s permanence over time at a suitable density in the plant tissues (4, 5). Plant probiotics can act directly by exerting PGP functions (i.e., biostimulation, biofertilization, and biocontrol) or their beneficial effect can be the result of interactions with the native endophytic community, whose composition can be modulated by the bacterial inoculum (6). The latter aspect is related to bacterial invasion, an ecological process that, in plant microbiology, has been mostly studied in terms of pathology (7). Nonetheless, a better understanding of beneficial bacteria establishment into the plant microbiome is a priority to overcome one of the major factors currently limiting the use of PGP strains in the field, namely their effective colonization of the holobiont (4).

In this study, we took advantage of *in vitro* micropropagated plants, limiting the variability of other experimental systems, to disentangle the effects played by putative beneficial bacteria when invading the plant holobiont. Micropropagated plants could be a useful tool to address the need for a wider portfolio of species for studying plant-microbiome interactions, including non-model species of relevant interest in agriculture (8). Though in the past bacterial occurrence in micropropagated plants was considered detrimental, more recently it has been clarified that certain bacteria can have beneficial effects on the explants in culture and can improve the micropropagation of recalcitrant genotypes (9). The grapevine plantlets used in this study were generated by somatic embryogenesis, providing virus-free plants (10), whose endophytic bacterial microbiota was characterized here for the first time. This simplified system was inoculated by bacteria isolated from field-grown grapevine or lettuce and identified as putative PGP strains through *in vitro* tests. The response of the endophytic microbiota associated with micropropagated grapevine to bacterial invasion was assessed by high throughput 16S rRNA amplicon sequencing, diversity, and network analyses.

## MATERIALS AND METHODS

### Isolation of endophytic bacterial strains, taxonomic identification, and screening for plant-growth-promoting traits

A collection of endophytic bacteria was established from the roots and leaves of *Lactuca sativa* (var. Canasta) and from the roots of the rootstock SO4 (*Vitis berlandieri* × *Vitis riparia*) grown in field conditions in a commercial vineyard of cv. Barbera (*Vitis vinifera*). The detailed isolation procedure is described in Supplementary Method 1. Bacterial isolates were identified through 16S rRNA gene amplification and partial sequencing (Macrogen, South Korea) as previously described (11). *In vitro* screening for PGP potential was performed for the entire bacteria collection. Inorganic phosphate solubilization and the production of indole-3-acetic acid (IAA), ammonia, protease, and exopolysaccharides (EPS) were assessed as described by Cherif and colleagues (12); 1-aminocyclopropane-1-carboxylic acid (ACC) deaminase activity was evaluated according to reference (13). The strains were also tested for *in vivo* PGP activity on lettuce (*L. sativa* isolates) and tomato (*Vitis* spp. isolates) using germination pouches (14). The promotion of growth parameters was assessed by comparing measures of the inoculated plants with those of untreated control plants using ANOVA and Dunnett’s test in the R software 4.2.0 (15). According to the taxonomic affiliation and PGP potential, two beneficial bacteria (*Rhizobium* sp. GR12 and *Kosakonia* sp. LL04VRBA) were selected for genetic manipulation and inoculation of micropropagated grapevine cuttings. The choice was oriented toward strains presenting multiple capabilities, favoring those displaying higher biostimulation (i.e., IAA production and ACC-deaminase activity), colonization (EPS production), and *in vivo* PGP potential.

### Fluorescent labeling of selected bacterial strains through conjugation

Bacterial strains were selected according to their PGP potential and genetically manipulated via conjugation with genes encoding for fluorescent proteins. Chromosomal insertion of the green fluorescent protein (GFP) in *Rhizobium* sp. GR12 was achieved utilizing the mini-Tn7 transposition system with the delivery plasmid pBK-miniTn7-*gfp3* (16). *Kosakonia* sp. LL04VRBA (referred to as VR04) was genetically modified via stable insertion of the gene encoding for the red fluorescent protein mScarlet-I (i.e., mSc) using the plasmid pMRE-miniTn7-145 as previously described (17). Tn7 insertions occur in specific attachment sites (attTn7), downstream of the coding region of the *glmS* gene and have been shown to pose no fitness cost to the bacterial host (16, 17).

### Inoculation of *in vitro* micropropagated grapevine and assessment of growth promotion activity under different conditions

Somatic embryogenesis was induced from immature anthers of *V. vinifera* cv. Chardonnay as previously described (18). The plantlets regenerated from somatic embryos were micropropagated by subculturing apical cuttings on half strength of Murashige and Skoog mineral salts (1/2 MS) without plant growth regulators and maintained in *in vitro* conditions (10). Strains *Rhizobium* sp. GR12-GFP and *Kosakonia* sp. VR04-mSc were grown in Luria-Bertani (LB) medium (Merck) at 30°C for 24 h. Bacterial cells were pelleted at 4,000 rpm for 10 minutes, then washed once and resuspended with 1/2 MS liquid medium. The strains were added at a final concentration of 10^6^ cells/mL in 200 mL of autoclaved MS medium supplemented with Agar type E 8 g/L (Merck) cooled at 50°C, in sterile glass jars. Grapevine cuttings before rooting were transferred to the jars in sterile conditions once the medium was solidified. The experiment was set up with two different media, 1/2 MS medium supplemented with vitamins (MSV) and MS medium without any supplement and diluted 1:20 (MSD), to grow plantlets under a nutritional deficit condition. To each condition, we applied three different treatments, i.e.*,* untreated negative control, *Rhizobium* sp. GR12-GFP and *Kosakonia* sp. VR04-mSc inocula. For each treatment, five replicate jars, containing five cuttings each, were set up and plants were let to take root and cultivated in a growth chamber (22°C, 55% humidity) for 30 days. Fresh weight was measured separately for root and shoot fractions after plant harvesting. Differences in biomass were assessed by applying analysis of variance (ANOVA) statistical test followed by Tukey multiple comparisons of means using R software version 4.2.0.

### Assessment of plant tissue colonization by inoculated strains

After plant harvesting, root and shoot fractions were stored at −80°C. Plant tissues were surface sterilized (Supplementary Method 2) and grounded in liquid nitrogen, then 0.1 g of samples were disrupted and homogenized with a TissueLyser II (Qiagen), and DNA was extracted with a DNeasy plant mini kit (Qiagen) according to the manufacturer’s instructions. Endophytic colonization of roots and shoots by the inoculated strains was assessed by quantitative PCR (qPCR) of the marker genes encoding for the fluorescent proteins, using serial dilutions of the plasmids used for strain molecular tagging as internal standards. For *Rhizobium* sp. GR12-GFP, the *gfp* gene was amplified with primers GFP-F (5′-GAAGATGGAAGCGTTCAA-3′) and GFP-R (5′-AGGTAATGGTTGTCTGGTA-3′) applying the following thermal protocol: 3 minutes at 98°C, followed by 40 cycles of 40 seconds at 98°C, 30 seconds at 58°C, and 30 seconds at 72°C (19). For *Kosakonia* sp. VR04-mSc, the mScarlet-I gene was amplified with primers FWD_Tn5/7_gt (5′-ATGGTGAGCAAGGGCGAG-3′) and REV_Tn5/7_gt (5′-CAACAGGAGTCCAAGCTCAG-3′) according to the protocol: 3 minutes at 98°C, followed by 35 cycles of 15 seconds at 98°C, 30 seconds at 60°C, and 30 seconds at 72°C (17). All reactions were performed with 200 nM primer concentration and 2× SsoAdvanced Universal SYBRGreen Supermix (Bio-Rad) and run in a Bio-Rad CFX Connect Real-Time PCR Detection System. Given the adopted conjugation system, for both strains, the copy number of the marker genes corresponds to the number of bacterial cells. In addition, to verify the strains’ viability in the plant tissues, dedicated samples were used for re-isolation trials and others were observed at the confocal microscope Laser Scanning Nikon A1 immediately after the collection. For the GFP signal, the green emission was excited at 488 nm and emission was collected at 500–550 nm; for the red signal of mScarlet, the excitation was at 561 nm and the emission was collected at 570–620 nm.

### 16S rRNA amplicon sequencing and metataxonomic analysis

16S rRNA amplicon sequencing was obtained by applying Illumina MiSeq sequencing of the V3–V4 hypervariable regions (20) of the bacterial 16S rRNA gene at Macrogen Korea. Peptide nucleic acid clamps (Eurogentec, 0.75 µM per reaction) were used to minimize the amplification of DNA from the plant’s mitochondria and plastids according to reference (21).

The obtained sequences were processed and analyzed using QIIME2 version 2022.2 (22) software. The DADA2 workflow was followed to assemble the reads and to perform denoising, dereplication, and chimera filtering following the default settings (23). Reads with minimum 99% sequence identity were clustered, and representative sequences were picked and defined as amplicon sequence variants, i.e., ASVs (24). Representative ASVs were classified using the SILVA SSU reference database version 138 (25) and BLAST+, implemented by the QIIME2 plugin consensus-blast (26). The adequacy of sequencing depth was evaluated by performing rarefaction analysis on the number of features observed with a rarified depth of 90,000 reads and the number of steps and iterations at the QIIME2 default settings. Bray-Curtis distance matrix on the normalized (log transformed) ASV table was used to perform beta-diversity analyses. Significant differences in bacterial community composition according to the factors “time” (i.e*.,* plant developmental stage), “fraction,” and “treatment” were investigated by PERMANOVA and CAP after the implementation of PERMDISP analysis to test the homogeneity of the dispersion. Statistical analyses were conducted in PRIMER v. 6.1, PERMANOVA+ for PRIMER routine (27). To identify the bacterial taxa (classified at the family level) whose distribution was significantly influenced by the applied treatments, a differential abundance analysis was performed using a quasi-likelihood *F*-test, and the sequencing depth was standardized using the relative log expression method as implemented in the R package EdgeR version 3.11 (28, 29). Richness (i.e*.*, number of families), Shannon diversity, and Evenness indices were calculated using the PAST software (30).

Co-occurrence Network interference (CoNet v1.1.1.beta) (31, 32) was employed to identify significant co-occurrence patterns among the bacterial communities. To such aim, the ASV table data were imported into Cytoscape (v3.9.1) (33) through the CoNet app. The top 100 edges with the highest positive and negative values were selected and combined using the mean value through the union approach. Multi-edge scores were then shuffled row-wise at 100 permutations (for the randomization). The brown method (34) was utilized to merge node pairs, which were assigned via the *P*-values of the multi-edges. Unstable edges were removed, and a significance threshold of *P* < 0.05 was applied to determine the *q*-value (the corrected significance value). The edges were colored via their positive (co-presence; green) and negative (co-exclusion; red) association. Finally, the hierarchical algorithm and the NetworkAnalyzer (4.4.8) (35) in Cytoscape were employed to construct the topology of the network and identify key taxa with low betweenness centrality.

### Isolation of culturable endophytes associated with *in vitro* micropropagated grapevine plantlets

Endophytic bacteria were isolated before rooting from the cuttings of *V. vinifera cv*. Chardonnay micropropagated in sterile conditions. The cuttings were surface sterilized with 70% ethanol and 1% sodium hypochlorite as reported in Supplementary Method 2, without the sonication step. Three different pools, containing five plants and weighing 1 g each, were smashed with 9 mL of 0.9% physiological solution, and serial dilutions were plated onto R2A or TSB agar media. To improve the efficacy of endophytic bacteria isolation (36), both media were supplemented with an extract obtained by blending *V. vinifera* cutting tissues with distilled water (0.2 g/mL) and then sterilized by filtration. The final concentration of the extract in the isolation medium was 3 mL/L. The plates were incubated for 5 days at 30°C, and visible single colonies were streaked three times on the same media to assess the growth of pure culture. All isolates were identified through 16S rRNA gene amplification and partial sequencing as described in the “Isolation of endophytic bacterial strains, taxonomic identification, and screening for plant-growth-promoting traits” section above. The bacterial collection was then subjected to genotyping based on the amplification of the ribosomal internal transcribed spacer (ITS) region (11). *In vitro* screening for plant growth-promoting activities was performed on at least one representative isolate for each ITS profile as described above. A second isolation experiment was then carried out in the same way to confirm the identification of the endophytic culturable microbiota associated with micropropagated Chardonnay cuttings.

### Dual-test inhibition assay

To assess the potential inhibition effect of strains *Rhizobium* sp. GR12 and *Kosakonia* sp. VR04 toward the growth of endophytic bacteria isolated from micropropagated grapevines, we applied the method previously detailed by Maida and coauthors (37). The test was carried out on the same representative isolate characterized for PGP activities. Strains *Rhizobium* sp. GR12 or *Kosakonia* sp. VR04 were streaked across one-half of a TSA plate and incubated at 30°C for 48 h. Target strains were then streaked in six replicates on the other half of the plate perpendicular to the initial streak, and plates were further incubated at 30°C for 48 h. The antagonistic effect was indicated by the absence or reduction of the target strain’s growth in the confluence area. For growth comparison, each target strain was streaked on negative control plates without tester strains *Rhizobium* sp. GR12 and *Kosakonia* sp. VR04. The presence of the inhibition effect was rated on each observation as complete (3), strong (2), weak (1), or absence of inhibition (0), in agreement with Maida and coauthors (37). Figure S1 includes exemplificative images of the possible levels of inhibition detectable through this assay.

### Genome sequencing and analyses

The genome of *Rhizobium* sp. GR12 was sequenced using Illumina NovaSeq technology and the reads were assembled using SPAdes (38). The functional annotation was performed using the RAST server (39).

### Nucleotide sequences availability

The nucleotide reads generated through Illumina NGS were deposited in the NCBI SRA database under the BioSample accessions SAMN3321079-SAMN33210660 and BioProject ID PRJNA932750.

The 16S rRNA nucleotide sequences of bacterial strains isolated from grapevine and lettuce grown in field conditions were deposited in the NCBI GenBank database under accession numbers OQ944163–OQ944272. The sequences belonging to micropropagated grapevine’s isolates were deposited under accession numbers OQ943234–OQ943337.

The genome of *Rhizobium* sp. GR12 was deposited in NCBI under the BioSample accession SAMN35731720 and BioProject ID PRJNA983687.

## RESULTS

### Selection of putative beneficial endophytic bacteria

Sixty different endophytic strains were isolated from lettuce roots (LR, 8 isolates), leaves (LL, 15 isolates), and grapevine roots (GR, 27 isolates). The results of the taxonomic identification of all strains and PGP activities screening for representative isolates are reported in Table S1. The most spread PGP activities in the bacteria collection were those related to ACC-deaminase activity and IAA production, detected in 55% and 67% of the tested strains, respectively. Considering the strains isolated from lettuce separately, EPS production and phosphate solubilization were widely spread activities being present in 73% and 48% of the strains, respectively. *Rhizobium* sp. GR12, isolated from grapevine roots, was able to produce IAA and promote the growth of tomato plants in germination pouches and was one of the few grapevine isolates capable of EPS production. *Kosakonia* sp. LL04VRBA (referred to as VR04) was isolated from lettuce leaves and tested positive for phosphate solubilization, EPS production, ACC deaminase activity, and IAA production. Moreover, VR04 was the only isolate out of 60 that significantly improved all plant growth parameters measured during the germination pouches assay (Table S1). These two bacteria were therefore selected for micropropagated plantlets’ inoculation. The molecular tagging of the two strains was successfully achieved, obtaining *Rhizobium* GR12 and *Kosakonia* VR04 strains able to express the GFP and mScarlet fluorescent proteins, respectively. The genetic manipulation did not have a significant effect on the growth of the two bacteria (Table S2).

### Endosphere colonization and growth promotion of *in vitro* grapevine plantlets by bacterial inoculants

**Fig 1:**
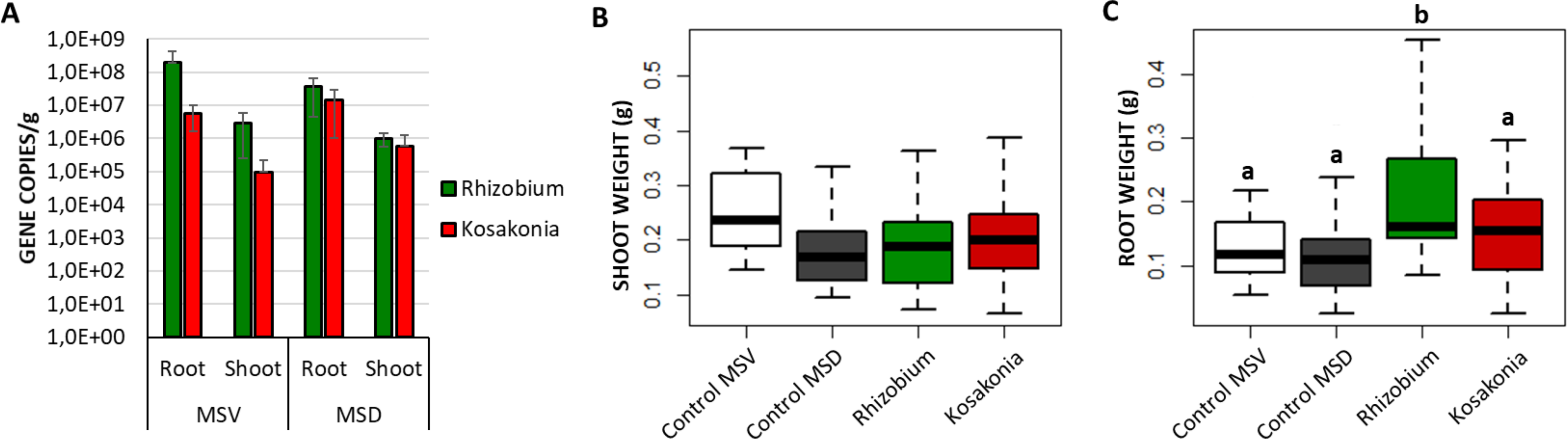
Colonization ability and PGP effect of *Rhizobium* sp. GR12-GFP and *Kosakonia* sp. VR04-mSc inoculated in micropropagated grapevine plantlets grown in optimal condition (MSV) or mimicking a condition of nutritional deficit (MSD). (**A**) Bar charts of qPCR results showing the copy number of the marker genes associated with the two bacteria in the endosphere of root and shoot fractions. The gene copy number corresponds to the strain cell number. (**B**) Plantlets’ shoot and (C) root fresh weight measured after 3 weeks of bacteria administration. Letters represent statistically significant differences according to ANOVA.

At the end of the inoculation experiment performed using *Rhizobium* sp. GR12-GFP or *Kosakonia* sp. VR04-mSc, the plant biomass was measured to evaluate the PGP effect of the two strains, and the colonization of the root and shoot endosphere was assessed through qPCR amplification of the marker genes from the total DNA extracted from plant tissues. Through observations at the confocal microscope, we assessed that the cells of both strains were viable and emitted fluorescence within the plant tissues of treated plants (Fig. S2). Results of qPCR showed that the tagged bacteria successfully established into the endosphere of both root and shoot fractions, though higher gene copy numbers were detected in the roots (Fig. 1A). Average values of marker genes varied between 10^6^ gene copies/g in the shoot and 10^8^ gene copies/g in the root tissues for plants inoculated with *Rhizobium* sp. GR12-GFP, and between 10^5^ gene copies/g and 10^7^ gene copies/g in plants colonized by *Kosakonia* sp. VR04-mSc (Table S3A). The specificity of the primers was assessed using the DNA of control plants as a template, and while amplification was absent with mSc primers, we observed a background signal up to 10^2^ copies/µL with GFP primers. However, there was no significant difference in the copy number of the two marker genes amplified in the plant tissues (ANOVA, *P* > 0.05), indicating a similar rate of colonization by the two strains under both growth conditions (Fig. 1A). Control plants grown on diluted medium displayed reduced development of the shoot compared to those grown under optimal conditions (Fig. 1B), though the difference was not statistically significant (*P* = 0.058; Table S3B). Strain *Rhizobium* sp. GR12-GFP promoted the development of the root system of grapevine plantlets grown under conditions of nutritional depletion by 40% when compared to the control group in the same conditions (*P* = 0.001; Fig. 1C; Table S3B). The annotated genome sequence of *Rhizobium* sp. GR12 showed the presence of functions related to stress response and siderophore production for iron assimilation, besides those responsible for auxin biosynthesis. In addition, functions possibly involved in plant colonization were detected (Table S4).

### Endophytic bacterial communities associated with *in vitro* grapevine plantlets and their interactions with invading bacteria

**Fig 2:**
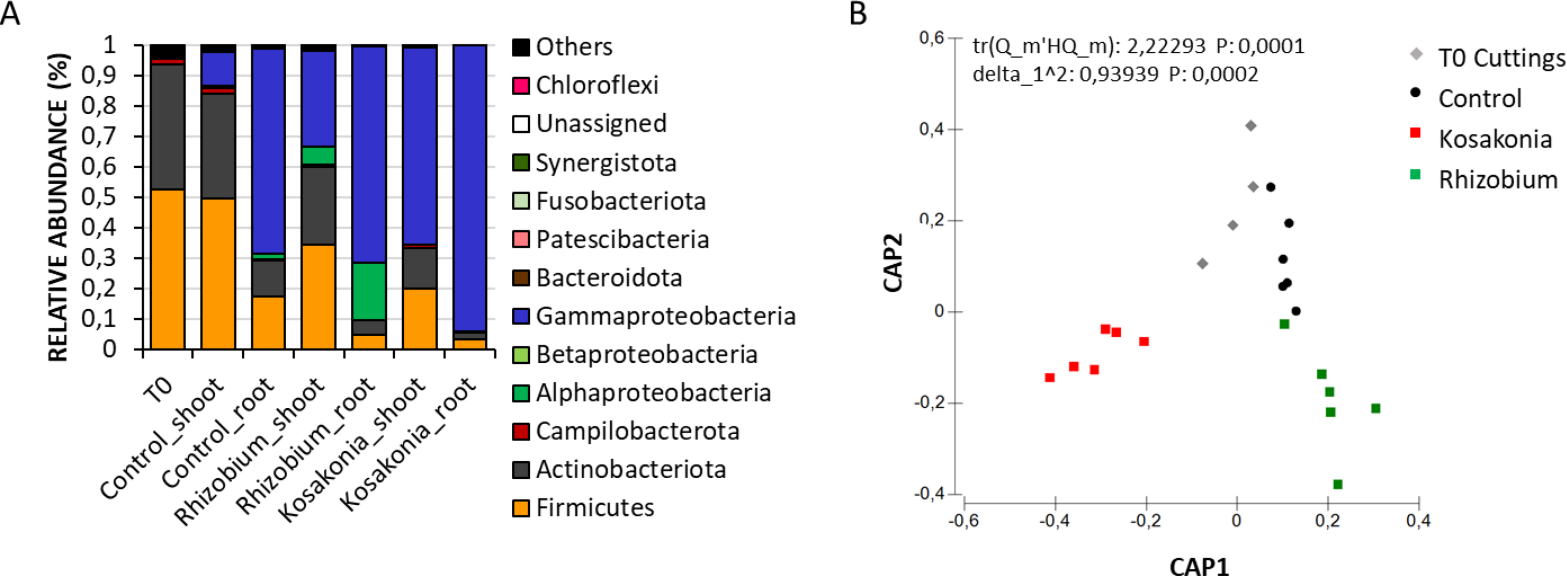
Taxonomy and diversity of endophytic bacterial community in control and inoculated micropropagated grapevine plantlets. (**A**) Bar chart analysis showing the relative abundance of the main phyla associated with T0 cuttings and with the root and shoot fractions of plantlets subjected to different inoculation treatments. (**B**) Canonical analysis of principal coordinates (CAP) showing the clustering of bacterial communities according to plant developmental stage and inoculation treatment.

A total of 3,648,071 high-quality sequences were obtained by sequencing the 16S rRNA amplicon libraries generated from the grapevine cuttings and the control and inoculated plantlets grown under nutritional deficit conditions. The average number of sequences per sample was 130,288, and the average sequence length was 406 ± 32 bp. Rarefaction curves were calculated and showed that the sequencing effort was adequate to cover the diversity within each sample (Fig. S3). Taxonomy assignment resulted in a data set of 675 unique ASVs (Table S5A). The endophytic microbiota of micropropagated grapevine cuttings (i.e., T0 samples) was dominated by Firmicutes (comprising between 45% and 58% of the ASVs detected at T0) and Actinobacteriota (37%–49%) (Table S5B; Fig. 2A). The more abundant families within these phyla were Streptococcaceae and Micrococcaceae, respectively, representing together over 60% of the total bacterial community of the grapevine cuttings. Veillonellaceae made up 6.5% of the community on average, while less abundant families were Actinomycetaceae (3.7%), Carnobacteriaceae (2.7%), and Pseudonocardiaceae (2.3%) (Table S5C). Proteobacteria represented on average 2% and Campylobacterota 1.7% of the bacterial community in cuttings (Table S5B).

At the end of the experiment, 1 month after rooting induction, we observed an increase in Proteobacteria in both control and inoculated plantlets. In control plantlets, this variation was attributed to a rise of reads associated with ASV3, classified as *Pantoea* within the family Erwinaceae, and it was mainly evident in the roots (Table S5A and C). A similar trend was observed in plantlets inoculated with *Rhizobium* sp. GR12-GFP, where, in addition to the ASV3 increase, ASV7 affiliated to the *Rhizobium* group appeared, representing between 12% and 24% of the total in the roots and from 2% to 11% in the shoots (Fig. S4A). In plantlets inoculated with *Kosakonia* sp. VR04-mSc, the Proteobacteria increase was attributable to ASV1 and ASV2 abundance, both Enterobacteriaceae affiliated to the genus *Kosakonia* (Table S5A and C). These two ASVs accounted for 87%–97% of the total endophytic community in the roots and between 45% and 89% in the shoots (Fig. S4B).

**Fig 3:**
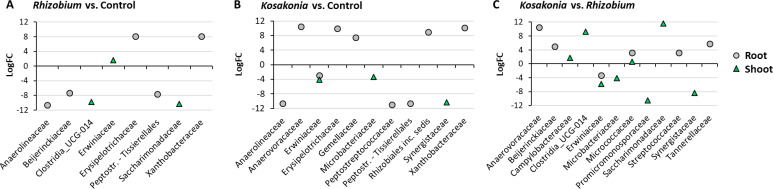
Differential abundance analysis results. The charts represent the fold changes (*Y* axis) in the abundance of bacterial families (*X* axis) that were significantly different (FDR-corrected *P*-value < 0.05) when comparing (A) *Rhizobium* treatment with control, (**B**) *Kosakonia* treatment with control, and (C) *Kosakonia* and *Rhizobium* treatments. Variations are represented separately for the root and shoot fractions.

To observe the effect of the applied treatments to the plantlet’s autochthonous microbiota, we then applied the differential abundance analysis to the data set considering the taxonomic level “family” and excluding the ASVs corresponding to the inoculated strains (i.e., ASV1, 2, and 7). The results showed a significant variation of eight taxa between the treatment *Rhizobium* and the control and of 11 taxa between the treatment *Kosakonia* and the control (Fig. 3). The analysis confirmed a statistically significant increase of Erwinaceae in the shoots and roots of plants treated with *Rhizobium* sp. GR12-GFP and in the control, compared to those inoculated with *Kosakonia* sp. VR04-mSc. In both inoculated treatments, we observed a substantial decrease of Anaerolineaceae and Peptostreptococcaceae in the root fraction compared to control plants, whereas Microbacteraceae were reduced only in the shoots of plantlets treated with *Kosakonia*.

No significant differences in terms of ASVs’ richness were detected among different treatments or plant fractions. On the other hand, ASVs’ diversity and ASVs’ evenness were significantly higher in the shoot than in the root fractions, with no variation among treatments (Fig. S5).

Following PERMDISP validation (*P* = 0.7096), the beta-diversity analysis on the ASVs’ data set showed that the bacterial communities of control plantlets significantly diverged from those of T0 cuttings, according to PERMANOVA main test (*P* = 0.0039). Bacterial communities were also significantly different in plantlets subjected to different inoculation treatments (PERMDISP, *P* = 0.2692; PERMANOVA main test, *P* = 0.0013), as confirmed by CAP analysis (Fig. 2B). Pair-wise test revealed that bacterial communities of T0 cuttings were significantly different from those of treated plantlets, while no significant difference was detected between T0 cuttings and control plantlets (Table S6). We tested by CAP analysis the effect of treatments on bacterial composition, considering each fraction separately: the bacterial communities of inoculated and control plants were significantly diverse in the root [tr(Q_m'HQ_m) = 1.90681; *P* = 0.0041] but not in the shoot fraction [tr(Q_m'HQ_m) = 1.82498; *P* = 0.1124].

Data summarizing the main interactions found with the network analyses are reported in Table S7. The network is characterized by 32 nodes and 214 edges, while the network density value is 0.216 and the clustering coefficient is 0.387. ASVs’ interactions considering the entire data set (Table S7A) are characterized by the prevalence of co-presence interactions (*n* = 204), with a few mutual exclusions (*n* = 10) representing the interplay of ASV1 and ASV2 with other ASVs, including ASV3 that corresponds to *Pantoea* sp. In control plantlets, the analysis showed that the simplified bacterial communities were characterized exclusively by ASVs co-occurrence (*n* = 101) while no mutual exclusion was detected (Table S7B; Fig. S6A). The network calculated referring only to control plants is characterized by 24 nodes and 101 edges, while the network density value is 0.183 and the clustering coefficient is 0.345. This peculiar situation was maintained after the establishment of *Rhizobium* sp. GR12-GFP (*n* = 101) into the plant tissues (Table S7C; Fig. S6B), displaying a network with similar characteristics (23 nodes, 101 edges, network density 0.2, and clustering coefficient 0.35). On the other hand, plant tissue invasion by *Kosakonia* sp. VR04-mSc caused a dramatic change in the ASVs’ interaction, and only mutual exclusion was detected (*n* = 117), apart from the observed co-occurrence between ASV1 and ASV2, both corresponding to the administered strain (Table S7D; Fig. S6C). The impact of *Kosakonia* sp. VR04-mSc is also summarized in the network features (37 nodes and 118 edges), which displayed a density of 0.089 and a clustering coefficient of 0.026. In control and *Rhizobium* treatments, the most connected nodes were affiliated to the bacterial families Streptococcaceae (16% and 18% of the total degree of connection, respectively), while Lactobacillaceae (9% of the total degree of connection), Staphylococcaceae (8%), Flavobacteriaceae (8%), and Anaerovoraceae (7%) mainly shaped the topology of the bacterial network in *Kosakonia-*treated plantlets.

### Characterization of the culturable microbiota from the endosphere of *in vitro* micropropagated grapevine cuttings

**Fig 4:**
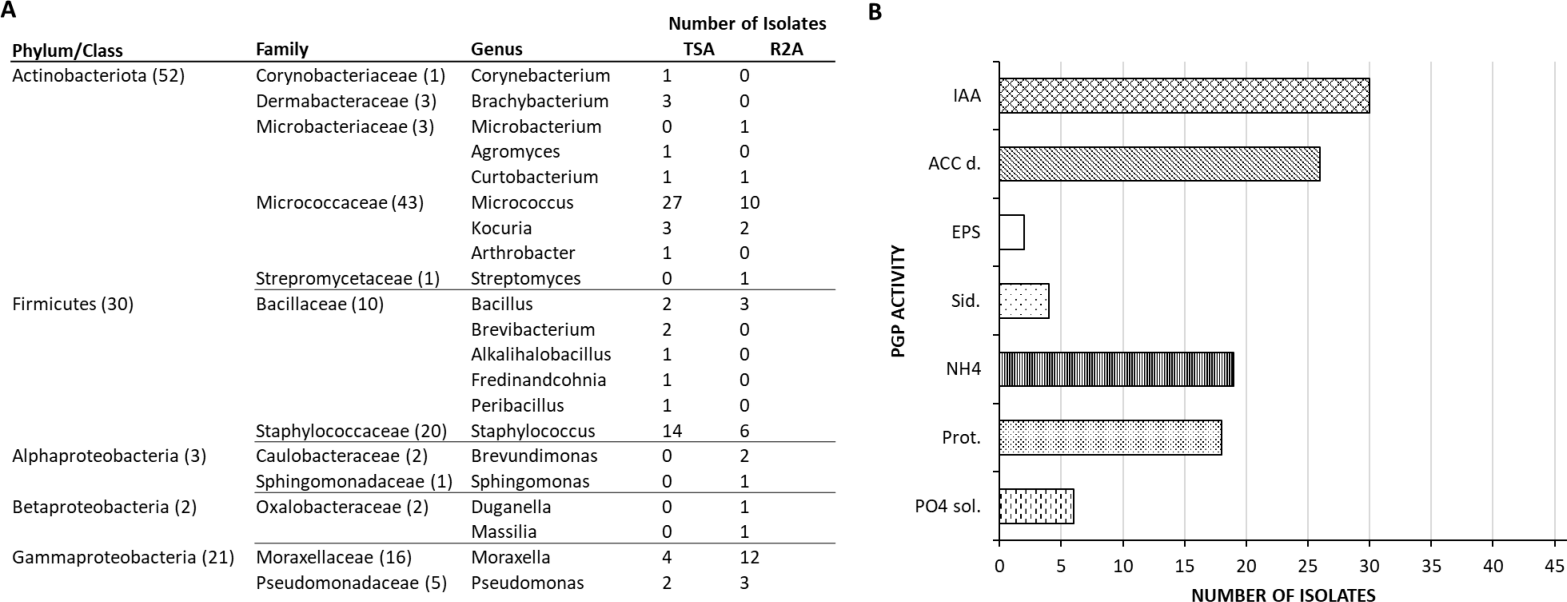
Taxonomical identification and PGP characterization of the bacterial collection isolated from the endosphere of micropropagated grapevine cuttings. (**A**) For each bacterial genus, the number of isolates obtained on TSA or R2A media is reported. Numbers in brackets indicate the total number of strains for each family and phylum/class. (**B**) The bar chart indicates the number of isolates that displayed positive results for each of the tested PGP activity. IAA, indole-acetic acid production; ACC d, ACC-deaminase activity; EPS, exopolysaccharide production; Sid, siderophore production; NH_4_, ammonia production; Prot, protease production; and PO_4_ sol, phosphate solubilization activity.

To further describe the microbiota of micropropagated grapevine cuttings, we also focused on the culturable fraction taxonomy and the potential growth inhibition caused by the administered strains. We isolated 105 endophytic bacteria from micropropagated grapevine cuttings (Table S8A), identifying 21 different genera. Actinobacteriota (Micrococcaceae) and Firmicutes (Staphylococcaceae) made up most of the collection, with the most abundant genera being *Micrococcus* (*n* = 37) and *Staphylococcus* (*n* = 20) (Fig. 4A). Gammaproteobacteria (Moraxellaceae and Pseudomonadaceae) were also abundant, with most of the isolates belonging to the genus *Moraxella* (*n* = 16). Within the dominant phyla, *Kocuria, Bacillus,* and *Pseudomonas* strains were also present (*n* = 5 per genus), while additional genera were more rarely cultured within Alpha- and Betaproteobacteria. During the second independent isolation experiment, performed to test the consistency of the isolation results, we randomly picked and identified 21 bacterial colonies (Table S8B). The results confirmed *Micrococcus* (*n* = 10), *Staphylococcus* (*n* = 3), *Moraxella* (*n* = 2), and *Microbacterium* (*n* = 2) as the most abundant culturable genera. The strains obtained during the first isolation were grouped into 48 ITS profiles, and one representative from each group was selected for *in vitro* screening of PGP activities (Table S8C). Biostimulation activities were spread through the collection with 30 representative strains being able to produce IAA and 26 displaying ACC deaminase activity (Fig. 4B). Protease and ammonia-producing bacteria were also abundant (*n* = 18 and *n* = 19, respectively), while few strains were able to produce siderophores (*n* = 4), EPS (*n* = 2), and solubilize inorganic phosphate (*n* = 6).

Finally, we investigated the antagonistic interactions occurring between *Kosakonia* sp. VR04 or *Rhizobium* sp. GR12 against the endophytic bacteria isolated from grapevine cuttings, revealing that 14 strains were sensitive to at least one of the two tester bacteria (Table 1). Most of the target strains displayed weak sensitivity (i.e., inhibition score = 1) to both *Kosakonia* sp. VR04 and *Rhizobium* sp. GR12. *Rhizobium* sp. GR12 was able to weakly inhibit all the tested strains. Considering the entire collection, only one of the tested interactions resulted in complete inhibition of the tested strain (i.e., isolate MG2-T24 belonging to the Microbacteriaceae family in the presence of *Kosakonia* sp. VR04).

**TABLE 1:** Dual-test inhibition assay results^*a*^

Strain	Family	NCBI identification	*Kosakonia*	*Rhizobium*
MG3-T5B	Bacillaceae	*Alkalihalobacillus clausii*	1	1
MG3-R9B	Bacillaceae	*Bacillus zanthoxyli*	2	1
MG3-T18B	Corynebacteriaceae	*Corynebacterium mucifaciens*	1	1
MG2-T24	Microbacteriaceae	*Agromyces humi*	3	2
MG3-T14	Microbacteriaceae	*Curtobacterium oceanosedimentum*	1	1
MG2-R17	Microbacteriaceae	*Curtobacterium oceanosedimentum*	0	1
MG2-R16	Microbacteriaceae	*Microbacterium aurum*	0	1
MG3-R6B	Moraxellaceae	*Moraxella osloensis*	1	1
MG2-R7B	Oxalobacteraceae	*Massilia timonae*	1	1
MG3-T5A	Pseudomonadaceae	*Pseudomonas luteola*	1	1
MG3-T11A	Pseudomonadaceae	*Pseudomonas oryzihabitans*	1	1
MG2-R7C	Pseudomonadaceae	*Pseudomonas oryzihabitans*	0	1
MG2-T25	Staphylococcaceae	*Staphylococcus epidermidis*	1	1
MG3-T6	Staphylococcaceae	*Staphylococcus epidermidis*	1	1

^*a*^ The table reports the endophytic strains isolated from micropropagated grapevine in which growth was inhibited by the presence of *Rhizobium* sp. GR12 or *Kosakonia* sp. VR04. Numbers indicate the level of inhibition: 0, absent; 1, weak; 2, strong; and 3, complete.

## DISCUSSION

In this work, we characterized the endophytic microbiota of *in vitro* cultivated *Vitis vinifera* var. Chardonnay plantlets, using this simplified system to study the outcomes of the bacterial invasion played by two putative beneficial bacteria on the holobiont, under controlled conditions.

The endophytic bacterial community of micropropagated grapevine cuttings was characterized both via high throughput 16S rRNA gene sequencing and cultivation approaches. The microbiota was dominated by taxa, such as Micrococcaceae, Streptococcaceae, Staphylococcaceae, Veillonellaceae, and Moraxellaceae, which are commonly detected and cultured from a broad range of environments including different plant tissues (40–42). However, this assembly showed to be radically different in terms of taxa’s relative abundance from those generally associated with *Vitis vinifera* cultivated in the field (43), even considering variations related to different rootstock-scion combinations (44, 45). Instead, it was more similar to indoor and human-associated microbiota (46, 47). The observed bacterial taxa, therefore, seem to be able to better adapt to the host plant under *in vitro* conditions and to replace, across generations of micropropagated plants, the dominating members of the native endophytic community, which were inherited from the parental plant tissue collected in the field. At the same time, plantlets obtained via somatic embryogenesis may recruit and maintain these taxa in the endosphere due to the establishment of a beneficial interaction. This was suggested by the detection of biostimulation activities, like ACC deaminase and the production of indole-acetic acid, in a high number of the strains isolated from cuttings. Even though the microbiota of *in vitro* cultured plants is poorly studied, evidence of stable association with PGP bacteria was found in other species such as strawberries (48) and papaya (49).

The invasion experiment performed with *Rhizobium* sp. GR12-GFP and *Kosakonia* sp. VR04-mSc demonstrated that the bacteria successfully colonized the endosphere of micropropagated grapevines cultivated on both standard and highly diluted growth media. Despite the fact that the two bacteria displayed PGP potential according to previous *in vitro* screenings, a growth promotion effect was only observed with *Rhizobium* sp. under simulated nutritional depletion. Bacteria within the rhizobia group are well known to associate with and support the growth of different plant hosts including grapevine (50). The sequence of the genome of strain *Rhizobium* sp. GR12, isolated from the root endosphere of field-grown grapevine, showed that this bacterium is endowed with several genes potentially involved in biofertilization and biocontrol (i.e., siderophores), stress response, and related to plant hormone regulation. Therefore, as we observed in this study under controlled conditions, the activation of root growth promotion may be an adaptive response to nutritional stress and not be manifested under optimal growth conditions, as previously demonstrated for drought stress (51).

The characterization of the bacterial microbiota of plantlets subjected to nutritional depletion revealed that the composition of the endophytic community was differently modulated by *Rhizobium* sp. GR12-GFP and *Kosakonia* sp. VR04-mSc, as specific taxa were enriched or depleted in response to the establishment of these bacteria, reflecting the different plant responses in terms of growth promotion. Considering the control plantlets as references, *Rhizobium* sp. GR12-GFP showed a minor impact on the plant bacterial community of recipient plantlets compared to *Kosakonia* sp. VR04-mSc. The different outcome is visible both considering the invader’s relative abundance over the total bacterial community and the variations of taxa composition. At the end of the experiment, we observed a drastic increase of ASV3, classified as *Pantoea* sp. within the family Erwinaceae, both in the controls and to a larger extent in plantlets inoculated with *Rhizobium* sp. GR12-GFP. This trend was not observed in the plantlets receiving the *Kosakonia* treatment, where the invader strain overcame all the other taxa in terms of relative abundance. *Pantoea* sp. is a well-known plant-associated bacterium with documented growth-promoting activities (52) and might have also played a role in sustaining micropropagated grapevine development in conditions of nutritional depletion. *Kosakonia* sp. VR04-mSc invasion caused a dramatic imbalance of the bacterial community structure as shown by co-occurrence network analysis. Such analysis showed the opposite outcome in terms of microbial interactions between the invader strain and the recipient endophytic community in *Kosakonia* sp. VR04-mSc or *Rhizobium* sp. GR12-GFP-inoculated plants. The highlighted negative or positive interactions among ASVs can also be explained by the indirect effects of the invader on the autochthonous microbial populations, as previously suggested (53). Indirect effects have been reported to be mediated by interactions with third species or by changes in any of the environmental factors, which drive the microbial community stability (54, 55). In the present study, considering the *in vitro* controlled conditions, we can hypothesize that the observed effects in terms of co-presence/mutual exclusion are most probably mediated by direct and third-part indirect interactions among the bacterial ASVs. Moreover, the results of the growth-inhibition test support the hypothesis that the invading strains activated mainly indirect interactions with the recipient community rather than a direct antagonist effect.

Modulation of the microbiota plays a key role in the capacity of the plant holobiont to balance nutrient acquisition. On the other hand, abiotic stress conditions such as starvation can make the plant more susceptible to opportunistic colonization (56, 57). In the present study, invasion by *Kosakonia* sp. VR04-mSc was associated with a dysbiosis of the endophytic microbiota that likely hampered the improvement of the host’s fitness under non-optimal conditions (58). All in all, besides a plant-growth-promoting effect directly exerted by *Rhizobium* sp. GR12-GFP, the improved performance of plants treated with this strain and their response to nutritional stress may also be related to the preservation of the microbial community structure and the holobiont’s functional integrity.

### Conclusions

This study showed that *in vitro* micropropagated *V. vinifera* plantlets host a microbiota assembly mostly composed of bacterial taxa commonly detected in indoor and human-associated environments, sharply divergent from that commonly found in grapevine endosphere under field conditions. By introducing two potential beneficial bacterial strains in this simplified ecosystem, we revealed the different outcomes of the invasion process toward the native endophytic bacterial populations, describing a relationship between the differential impact on community structure and the plant growth promotion in conditions of nutritional depletion. Overall, our results confirm the importance of preserving the native endophytic community structure and functions when attempting to engineer the plant microbiome (59).

Furthermore, the results generated by characterizing the cultured microbiota associated with grapevine cuttings and by the inoculation of *Rhizobium* sp. GR12 propose the possible exploitation of PGP bacteria for the biostimulation of *in vitro* plant cultures (60). This opens up a future research perspective for the reduction of chemical use and plant stress during the transplant phase.

## Data Availability

DNA sequencing data are available in the NCBI SRA and GenBank database, as detailed in the Materials and Methods section. Additional data (cited as Tables S1 to S3 and S5 to S7) have been deposited in the Dataverse repository (https://dataverse.unimi.it/dataset.xhtml?persistentId=doi:10.13130/RD_UNIMI/U1VCP7).

## SUPPLEMENTAL MATERIAL

Supplementary information aem.02078-23-s0001.docx Supplementary methods, tables, figures, and captions.

ASM does not own the copyrights to Supplemental Material that may be linked to, or accessed through, an article. The authors have granted ASM a non-exclusive, world-wide license to publish the Supplemental Material files. Please contact the corresponding author directly for reuse.
