# Supplementary material for: Deciphering the interaction of bacteria inoculants with the recipient endophytic community in grapevine micropropagated plants
Source: Appl Environ Microbiol. 2024 Jan 30;90(2):e02078-23. doi: 10.1128/aem.02078-23 (PMC10880630; doi:10.1128/aem.02078-23)
Supplement: Supplementary information — Supplementary methods, tables, figures, and captions. [file aem.02078-23-s0001.docx]

**Deciphering the interaction of bacteria inoculants with the recipient endophytic community in grapevine micropropagated plants**

Lorenzo Vergani^1^, Joa Patania^1^, Valentina Riva^1^, Luca Nerva^2,3^, Floriana Nuzzo^2^, Giorgio Gambino^2^, Sara Borin^1^, Francesca Mapelli^1^*

^1^Department of Food, Environmental and Nutritional Science (DeFENS), University of Milan, Milan, Italy

^2^Institute for Sustainable Plant Protection, National Research Council of Italy (IPSP-CNR), Turin, Italy

^3^Italy Research Centre for Viticulture and Enology, Council for Agricultural Research and Economics, Conegliano, Italy

^*^corresponding author: francesca.mapelli@unimi.it

**SUPPLEMENTARY METHODS**

**Supplementary Method 1**

For endophytic bacteria isolation, three replicates’ samples were collected and pooled for each plant species. *L. sativa* var. Canasta specimens were collected from an organic agricultural field in Lombardy (North Italy), while roots of rootstock SO4 (*Vitis berlandieri x Vitis riparia*) were collected from a vineyard of cv. Barbera located in Piedmont (North Italy). Plant tissues were rinsed and surface sterilized before isolation, as follows: i) *L. sativa* roots were cleaned from soil with 0.9% physiological solution, then washed with 70% ethanol for 3’, 0.7% sodium hypochlorite for 5’, 70% ethanol for 30’’ and five times with sterile distilled water; ii) *L. sativa* leaves were washed with 0.7% sodium hypochlorite and five times with sterile distilled water; iii) grapevine roots were cleaned from soil with 0.9% physiological solution, then washed with 70% ethanol for 3’, 1% sodium hypochlorite for 5’, 70% ethanol for 30’’ and five times with sterile distilled water. Effective surface sterilization was assessed by observing no bacterial growth after plating the last washing water on TSA medium. Tissues were grounded in a sterile mortar with sterile 0.9% physiological solution, then serial dilutions were plated on King’s B and VRB Agar media for *L. sativa* and on King’s B and R2 Agar media (Merck) for grapevine. Plates were incubated for 48 hours at 30°C, then visible single colonies were streaked three times on the same media to obtain pure cultures. Strains were stored at -80 °C in liquid media supplemented with 25% glycerol.

**Supplementary Method 2**

For the surface’ sterilization of micropropagated grapevine roots and shoots the following protocol was applied: three sonication cycles of 30” (UP100H ultrasonic processor, dr. Hielscher GmbH, amplitude 50%, continuous mode) in PBS buffer; plant tissues were transferred in clean tubes and washed with 70% ethanol for 3’, 1% sodium hypochlorite for 5’ and then five times with sterile distilled water. To verify the removal of epiphytic bacteria an aliquot of 100 µL water was collected from the last washing step and plated on LB plates (SIGMA) and, in addition, surface sterilized samples were observed using an epifluorescence microscope (Zeiss Axio Lab.A1). Samples were used for subsequent analyses when no colonies developed on LB plates from the 100 µL rinsing water and no fluorescent signals were observed on rhizoplane after sterilization.

**SUPPLEMENTARY RESULTS**

**Supplementary Table 1.** Taxonomic identification and PGP traits of endophytic bacteria isolated from grapevine and lettuce. Dark grey squares indicate positive results to *in vitro* PGP tests; green squares represent statistically significant promotion of growth parameters in the germination pouches test. The table is available in the Dataverse repository at the following link: <https://dataverse.unimi.it/dataset.xhtml?persistentId=doi:10.13130/RD_UNIMI/U1VCP7>.

**Supplementary Table 2.** Results of the growth experiment conducted on the wild-type and tagged strains *Rhizobium* sp. GR12 and *Kosakonia* sp. VR04 using TSB and MS media. **A)** Raw data, **B)** *Rhizobium* sp. GR12, **C)** *Kosakonia* sp. VR04. The table is available in the Dataverse repository at the following link: <https://dataverse.unimi.it/dataset.xhtml?persistentId=doi:10.13130/RD_UNIMI/U1VCP7>.

**Supplementary Table 3. A)** Results of quantitative PCR on the marker genes coding for GFP and mScarlet proteins. The table reports the starting quantity (SQ) for each PCR reaction and the corresponding number of gene copies expressed over gram of plant tissue. **B)** Measurements of plant fresh biomass at the end of the inoculation experiment. Raw data of the root and shoot fresh weight for each sample, and statistical analysis are reported. The table is available in the Dataverse repository at the following link: <https://dataverse.unimi.it/dataset.xhtml?persistentId=doi:10.13130/RD_UNIMI/U1VCP7>.

**Supplementary Table 4.** Genomic features of *Rhizobium* sp. GR12 and list of the metabolic functions possibly involved in plant colonization and plant growth promotion.

| **Genome Characteristics** |  | **GR12** |
| --- | --- | --- |
|  |  |  |
|  | Sequence size (bp) | 5,690,492 |
|  | Number of contigs | 65 |
|  | GC content (%) | 59.1 |
|  | CDS number | 5,687 |
| **Subsystem Feature Counts** |  |  |
|  |  |  |
| **Iron acquisition and metabolism** | Iron siderophores sensor and receptor system | 46 |
|  | Siderophores assembly kit | 23 |
|  | Siderophores aerobactin | 9 |
| **Secondary metabolism** | Plant hormones - Auxin biosynthesis | 4 |
| **Stress response** | Osmotic stress | 26 |
|  | Oxidative stress | 46 |
|  | Detoxification | 13 |
|  | Stress response | 6 |
|  | Periplasmic stress | 2 |
| **Motility and chemotaxis** | Flagellar motility in Prokaryota | 58 |
| **Virulence, disease and defense** | Bacteriocins, ribosomally synthesized and antibacterial peptides | 1 |
|  | Resistance to antibiotics and toxic compounds | 26 |
|  | Invasion and intracellular resistance | 13 |

**Supplementary Table 5. A)** Abundance of the different ASVs resulting from taxonomical classification at the genus level in each biological sample. **B)** Relative abundance (%) of bacterial taxa at phylum and **C)** family level in each sample. The table available in the Dataverse repository at the following link: <https://dataverse.unimi.it/dataset.xhtml?persistentId=doi:10.13130/RD_UNIMI/U1VCP7>.

**Supplementary Table 6.** PERMANOVA analysis on beta-diversity analysis based on ASVs relative abundance in micropropagated root and shoot samples. **A)** PERMANOVA main test of ‘Time’ (i.e., plant developmental stage) and ‘Fraction’ and ‘Treatment’ factors on ASVs distribution. **B)** Pair-wise tests. Significant effects are indicated by asterisks. Df: degrees of freedom; MS: mean sum of squares; Pseudo-F: F value by permutation; P: p statistic.

| **A. PERMANOVA table of results** | |  |  |  |  |  |  |
| --- | --- | --- | --- | --- | --- | --- | --- |
| Source | df | SS | MS | Pseudo-F | P(perm) | perms | P(MC) |
| time | 1 | 2938,8 | 2938,8 | 2,8533 | 0,0007 | 5436 | 0,0039 |
| Res | 20 | 20599 | 1030 |  |  |  |  |
| Total | 21 | 23538 |  |  |  |  |  |
| Source | df | SS | MS | Pseudo-F | P(perm) | perms | P(MC) |
| fraction | 1 | 3681,7 | 3681,7 | 3,7083 | 0,0001 | 9764 | 0,0005 |
| Res | 20 | 19857 | 992,83 |  |  |  |  |
| Total | 21 | 23538 |  |  |  |  |  |
| Source | df | SS | MS | Pseudo-F | P(perm) | perms | P(MC) |
| treatment | 3 | 6351,4 | 2117,1 | 2,2173 | 0,0001 | 9847 | 0,0013 |
| Res | 18 | 17187 | 954,82 |  |  |  |  |
| Total | 21 | 23538 |  |  |  |  |  |
| **B. PAIR-WISE TESTS** |  |  |  |  |  |  |  |
| Term 'treatment' |  |  |  |  |  |  |  |
| Groups | t | P(perm) | perms | P(MC) |  |  |  |
| Control, Rhizobium | 1,1852 | 0,0821 | 462 | 0,2073 |  |  |  |
| Control, T0 | 1,3239 | 0,0339 | 210 | 0,1274 |  |  |  |
| Control, Kosakonia | 1,3471 | 0,0405 | 462 | 0,0878 |  |  |  |
| Rhizobium, T0 | 1,7681 | 0,0057 | 210 | 0,0139 |  |  |  |
| Rhizobium, Kosakonia | 1,5479 | 0,0026 | 462 | 0,026 |  |  |  |
| T0, Kosakonia | 1,8096 | 0,0047 | 210 | 0,0137 |  |  |  |

**Supplementary Table 7.** Co-occurrence network analysis. Sheets A, B, C, D report the most important parameters calculated for the co-occurrence analyses considering the whole dataset together **(A)**, the control plants **(B)**, the plants inoculated with *Rhizobium* sp. GR12-GFP **(C)** and the plants inoculated with *Kosakonia* sp. VR04-mSc **(D)**. The type of interaction (copresence/mutual exclusion) and values of EdgeBetweenness, the merged p-value and q-value are reported for any significant interaction identified. Sheet **(E)** reports the percentage of nodes showing co-occurrence or mutual exclusion associations for each taxonomic family within the three treatments. The table is available in the Dataverse repository at the following link: <https://dataverse.unimi.it/dataset.xhtml?persistentId=doi:10.13130/RD_UNIMI/U1VCP7>.

**Supplementary Table 8.** Taxonomic identification of endophytic bacteria isolated from micropropagated grapevine plants during the first **(A)** and second **(B)** isolation experiments. **C)** PGP traits of the isolated strains. Dark grey squares indicate positive results to *in vitro* PGP tests. The table is available in the Dataverse repository at the following link: <https://dataverse.unimi.it/dataset.xhtml?persistentId=doi:10.13130/RD_UNIMI/U1VCP7>.

**Supplementary Figure 1.** Exemplification of the dual test inhibition assay results. The pictures show the inhibition of the growth of different target strains when inoculated against the tester strain VR04 (on the right), compared to a negative control (on the left). Each picture corresponds to a different level of inhibition: A) 0=absent, B) 1=weak, C) 2=strong, D) 3=complete.


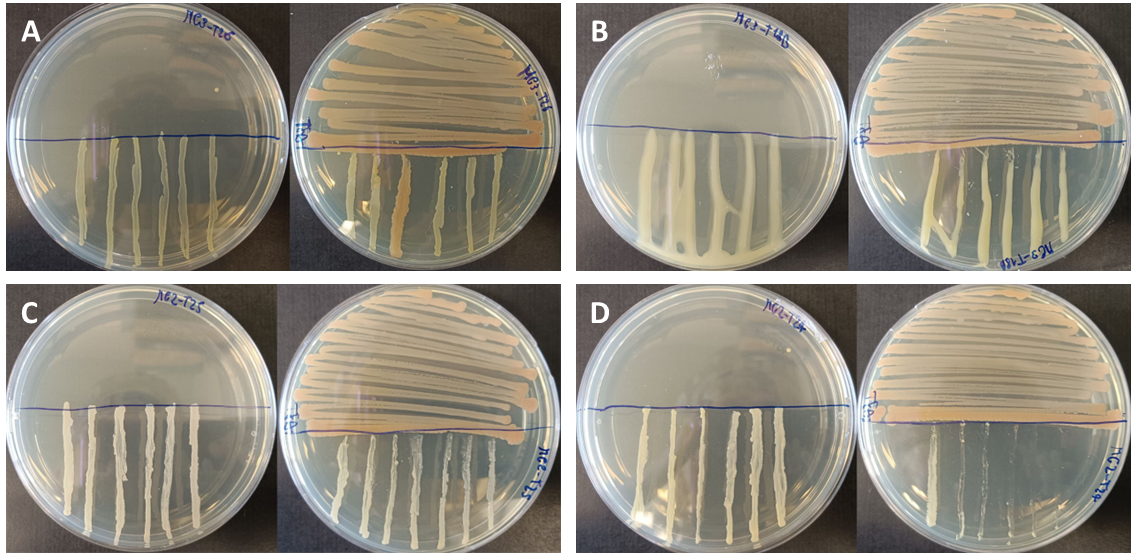


**Supplementary Figure 2.** Confocal microscopy images showing A) green fluorescent cells of *Rhizobium* sp. GR12 and B) red fluorescent cells of *Kosakonia* sp. VR04 within the tissues of micropropagated *Vitis vinifera* leaves. Blue indicates the chlorophyll signal.

**
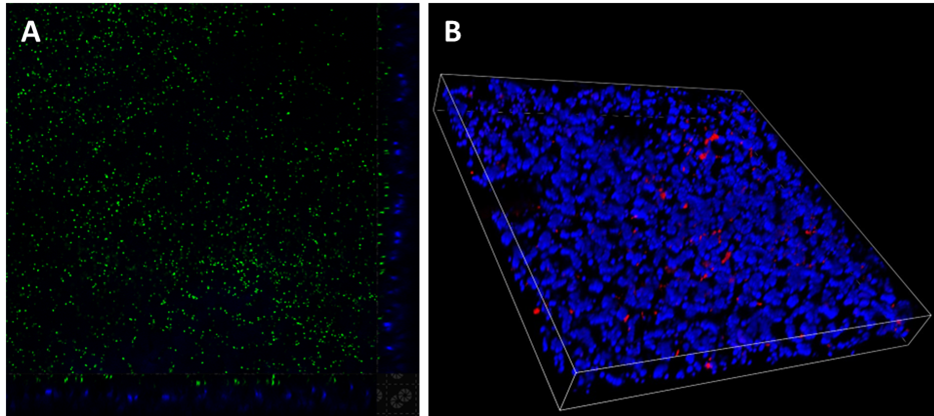
**

**Supplementary Figure 3.** Rarefaction curves of the 16S rRNA gene Illumina libraries. Rarefaction curves were calculated for each sample setting a sequencing effort cut-off on the basis of the samples with the lowest number of reads.


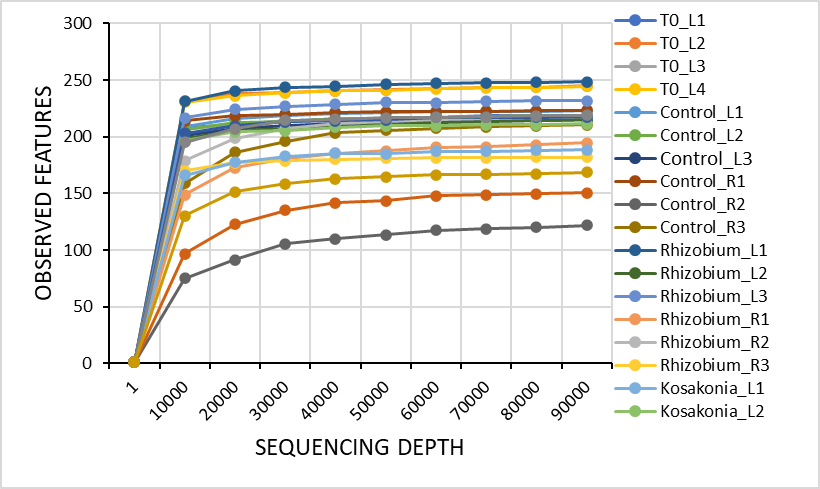


**Supplementary Figure 4.** Relative abundance of the ASVs corresponding to the genus of the inoculated strains on the total number of reads observed in the endophytic bacterial community of roots and shoots of micropropagated grapevines. A) Plantlets inoculated with *Rhizobium* sp. GR12. B) Plantlets inoculated with *Kosakonia* sp. VR04.

**
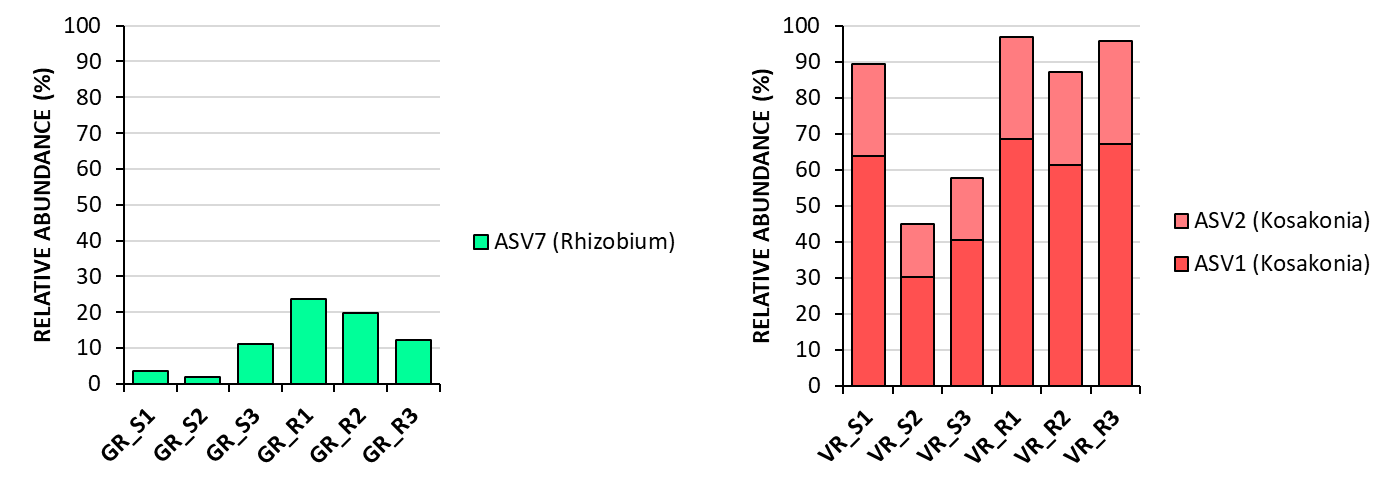
**

**Supplementary Figure 5.** Alpha diversity analysis of bacterial communities of the different inoculation treatments and plant fraction. A) ASV Richness, (B) ASV diversity according to Shannon index and (C) ASV evenness. Statistically significant differences (ANOVA, p<0.05) according to the factor “treatment” are indicated by letters.

**
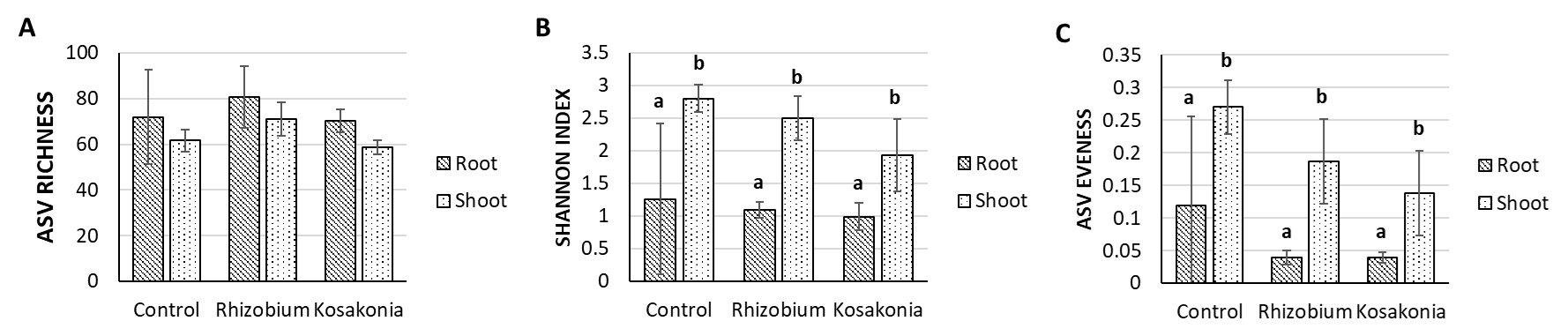
**

**Supplementary Figure 6.**

Significant co-occurrence and mutual exclusion interactions between ASVs as detected by the network analysis. Interaction among ASVs in the A) Control, B) Rhizobium and C) Kosakonia treatments.

**
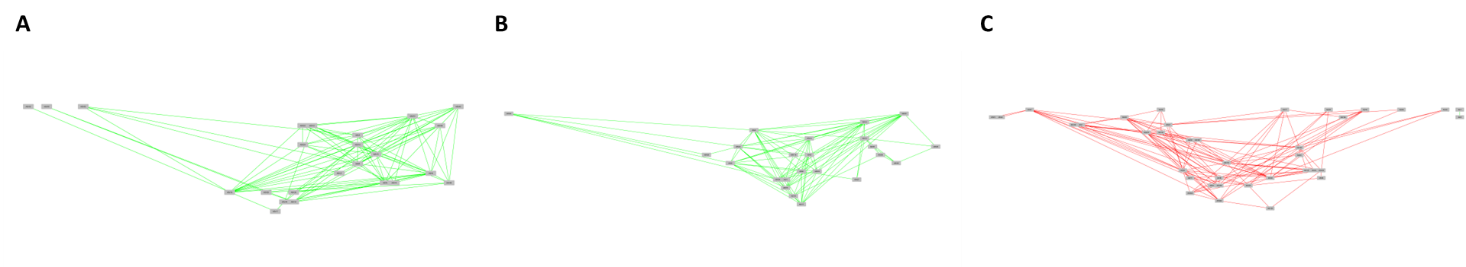
**
